# Psychological flexibility as a mechanism of change in psilocybin-assisted therapy for major depression: results from an exploratory placebo-controlled trial

**DOI:** 10.1038/s41598-024-58318-x

**Published:** 2024-04-17

**Authors:** Jordan Sloshower, Richard J. Zeifman, Jeffrey Guss, Robert Krause, Hamideh Safi-Aghdam, Surbhi Pathania, Brian Pittman, Deepak Cyril D’Souza

**Affiliations:** 1 Department of Psychiatry, Yale University School of Medicine, New Haven, CT USA; 2 Psychiatry Service, VA Connecticut Healthcare System, West Haven, CT USA; 3 West Rock Wellness PLLC, New Haven, CT USA; 4 NYU Langone Center for Psychedelic Medicine, NYU Grossman School of Medicine, New York, NY USA; 5 Centre for Psychedelic Research, Division of Psychiatry, Department of Brain Sciences, Faculty of Medicine, Imperial College London, London, UK; 6 Department of Psychiatry, NYU Grossman School of Medicine, New York, NY USA; 7 Yale School of Nursing, New Haven, CT USA; 8 Centered PLLC, New Haven, CT USA

**Keywords:** Outcomes research, Depression, Combination drug therapy

## Abstract

Several phase II studies have demonstrated that psilocybin-assisted therapy shows therapeutic potential across a spectrum of neuropsychiatric conditions, including major depressive disorder (MDD). However, the mechanisms underlying its often persisting beneficial effects remain unclear. Observational research suggests that improvements in psychological flexibility may mediate therapeutic effects. However, no psychedelic trials to date have substantiated this finding in a clinical sample. In an exploratory placebo-controlled, within-subject, fixed-order study, individuals with moderate to severe MDD were administered placebo (n = 19) followed by psilocybin (0.3 mg/kg) (n = 15) 4 weeks later. Dosing sessions were embedded within a manualized psychotherapy that incorporated principles of Acceptance and Commitment Therapy. Depression severity, psychological flexibility, mindfulness, and values-congruent living were measured over a 16-weeks study period. Psychological flexibility, several facets of mindfulness, and values-congruent living significantly improved following psilocybin and were maintained through week 16. Additionally, improvements in psychological flexibility and experiential acceptance were strongly associated with reductions in depression severity following psilocybin. These findings support the theoretical premise of integrating psilocybin treatment with psychotherapeutic platforms that target psychological flexibility and add to emerging evidence that increasing psychological flexibility may be an important putative mechanism of change in psilocybin-assisted therapy for MDD and potentially, other mental health conditions.

## Introduction

In recent years, there has been a revitalization of interest in the therapeutic potential of psychedelic substances for a variety of neuropsychiatric and behavioral health conditions^1,2^. Psilocybin, a naturally occurring alkaloid found in the *Psilocybe* genus of mushrooms, is categorized as a “classical psychedelic” compound along with lysergic acid diethylamide (LSD), dimethyltryptamine (DMT), and others. These drugs produce a range of acute perceptual and mood alterations in humans presumably through agonism of brain serotonin 5HT-2A receptors^3,4^.

In the past decade, a number of early phase trials have suggested that psilocybin-assisted therapy (i.e., embedding psilocybin dosing sessions within a framework of psychological support or psychotherapy) may have rapid acting and persisting therapeutic effects across a number of mental health conditions, including depression, anxiety, and substance use disorders^5–14^. These beneficial effects have been reported after just one or two doses of psilocybin, and in some cases, may persist up to 1–4.5 years post-administration^15,16^.

However, the mechanisms by which psilocybin-assisted therapy may produce both rapid and sustained antidepressant effects remain unclear. A number of neurobiological, psychological, cognitive/behavioral and spiritual mechanisms have been proposed to explain psilocybin-assisted therapy’s rapid and sustained antidepressant effects. For instance, certain psilocybin treatment studies have found that positive clinical outcomes were correlated with or partially-mediated by mystical-type subjective experiences^12–14,17,18^ or alterations in neuroplasticity^19^. Other potential psychological and cognitive/behavioral mechanisms include increased psychological flexibility and experiential acceptance^20–22^, increased connectedness^23,24^, relaxation and revision of high-level beliefs^25,26^, and emotional breakthrough^27^.

Given that multiple potential mechanisms may contribute to the therapeutic effects of psilocybin-assisted therapy, it remains unclear which psychotherapeutic models and approaches are optimal. Some studies have employed non-specific supportive psychotherapeutic models while others have incorporated elements of evidence-based, condition-specific therapies. In developing a therapeutic approach for an exploratory clinical trial of psilocybin-assisted therapy for major depression, our group sought an evidence-based therapy that would be synergistic with the subjective effects of psilocybin and offer a structure for the preparation and integration psychotherapy sessions. After evaluating a range of potential therapies, we selected Acceptance and Commitment Therapy (ACT) given conceptual and phenomenological overlaps between ACT and psilocybin-assisted therapy^20^.

Within Acceptance and Commitment Therapy, psychological flexibility is conceptualized as the central organizing principle underlying mental well-being and therapeutic change^28^. Psychological flexibility is a multifaceted construct composed of: (a) openness to experience (experiential acceptance); (b) behavioral awareness (i.e., mindful attention to the present moment); and (c) values-driven action^29^. In line with this model, global psychological inflexibility and several of its subcomponents (most notably experiential avoidance) have been linked with depression severity (for reviews, see^30,31^) and improvement in these measures linked with clinical improvement following ACT^32–35^.

### Psychedelics and psychological flexibility

A growing body of research suggests that psychedelic therapy may increase psychological flexibility and lead to therapeutic benefits via these changes. Several cross-sectional and observational studies have found that use of a classic psychedelic is associated with subsequent increases in psychological flexibility^21,36–38^. Furthermore, following classic psychedelic use, increases in psychological flexibility have been found to be associated with improvements in mental health, including depressive symptoms^22,36^, anxious/depressive symptoms^21^, positive and negative affect^37^, and grief^38^. These findings suggest that psychological flexibility may be a transdiagnostic mechanism contributing to the positive effects of psychedelics. Importantly, research has not yet examined the effect of psilocybin-assisted therapy on psychological flexibility (or the effect of any psychedelic on psychological flexibility) within a clinical sample. Furthermore, no clinical trials to date have examined the association between increases in psychological flexibility and improvements in depressive symptoms following psilocybin-assisted therapy.

Mindfulness (defined as awareness and acceptance of one’s inner experiences)^39^ is closely related to several components of psychological flexibility, including behavioral awareness and experiential acceptance^29,40^. In a sample with MDD, a recent randomized controlled trial found that, relative to escitalopram, psilocybin-assisted therapy led to greater increases in experiential acceptance^5^. Furthermore, within this trial, increases in experiential acceptance following psilocybin-assisted therapy predicted improvements in depression severity, anxiety, suicidal ideation, and well-being^41^. Within non-clinical samples, several studies have observed increases in facets of mindfulness (most consistently the facets of experiential acceptance/non-judgmental awareness) following use/administration of classic psychedelics^22,42–49^, including psilocybin^50–52^ (for a review, see^53^). Furthermore, in observational studies, increases in non-judgment of inner experience (closely related to experiential acceptance) following psilocybin were associated with reductions in anxiety^50^. However, no clinical trials have examined the effect of psilocybin-assisted therapy on mindfulness or its association with treatment outcomes.

Several naturalistic studies have found that classic psychedelic use is associated with changes in values. Among individuals that reported decreases in smoking following classic psychedelic use, participants attributed changes in life priorities/values as the primary psychological factor that contributed to this behavior change^54^. Research has also found that administration of psilocybin alongside support for spiritual practice is associated with increases in valuing of tradition^55^. However, the effect of classic psychedelics on values has not yet been examined in the context of a psychedelic clinical trial or within a clinical sample.

In sum, psychological flexibility is a promising putative transdiagnostic mechanism underlying psychotherapeutic interventions and potentially, the beneficial effects of psychedelic therapy. However, to date, research has not examined the effect of psilocybin-assisted therapy on (a) psychological flexibility; (b) mindfulness; or (c) values within a clinical trial. Furthermore, clinical research has not yet examined whether psilocybin-assisted therapy leads to improvements in depression severity via its effects on psychological flexibility. Finally, research has not yet evaluated the effect of psilocybin on psychological flexibility when psilocybin is combined with a therapeutic intervention specifically designed to increase psychological flexibility. Therefore, using data from a recent clinical trial of psilocybin-assisted therapy that used ACT as a therapeutic frame (for primary clinical outcomes, see^11^), we examined (1) the effects of psilocybin-assisted therapy on (a) psychological flexibility/experiential acceptance, (b) mindfulness, and (c) values-congruent living, as well as (2) the association between changes in psychological flexibility/experiential acceptance and reductions in depression severity.

## Methods

This study was conducted with approvals from the Institutional Review Boards of the VA Connecticut Healthcare System and Yale University, under a US Food and Drug Administration-approved Investigational New Drug application (D’Souza# IND #124,874), under a schedule 1 license from the US Drug Enforcement Agency (DEA) and was registered on clinicaltrials.gov on 13/06/2018 (NCT03554174). The authors assert that all procedures contributing to this work comply with the ethical standards of the relevant national and institutional committees on human experimentation and with the Helsinki Declaration of 1975, as revised in 2008.

### Study design

**Figure 1:**
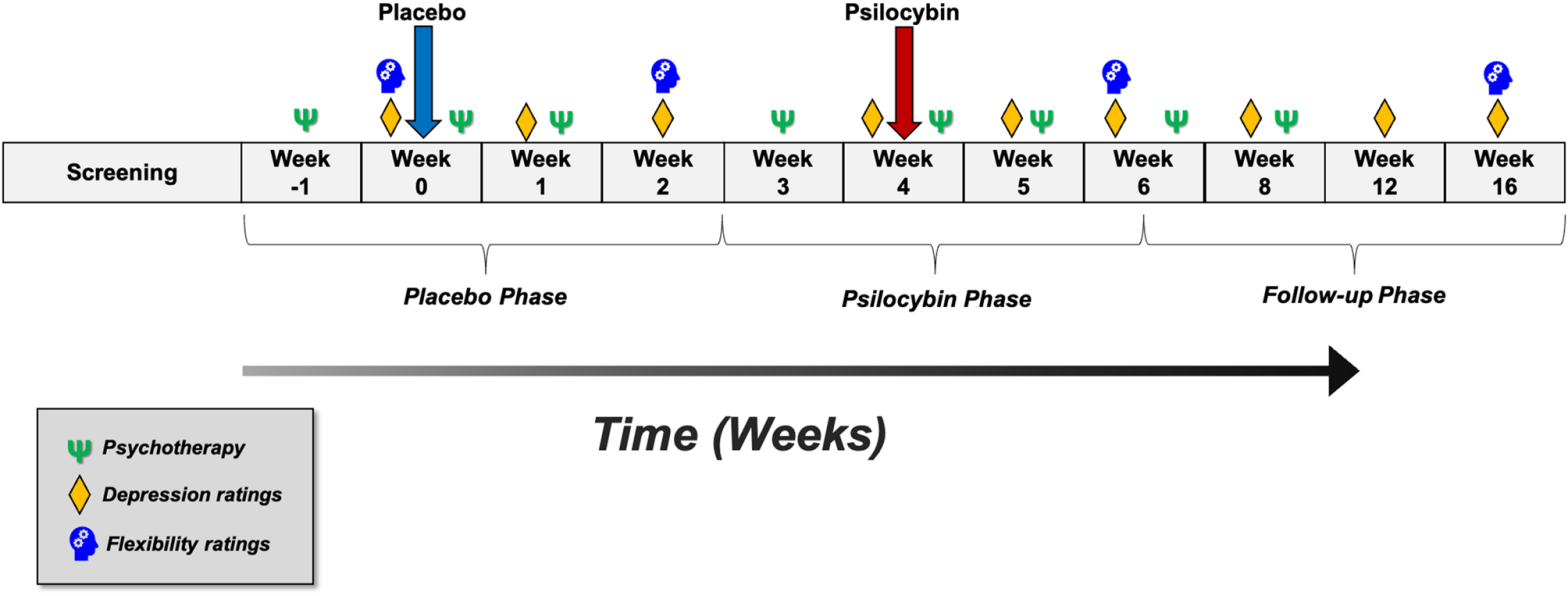
Study design and flow.

The parent study from which the data presented here was derived utilized a placebo-controlled, within-subject, fixed-order design with enhanced blinding procedures. This study was designed primarily to investigate psilocybin’s effects on an electrophysiological biomarker of neuroplasticity (see^19^), rather than to demonstrate changes in clinical or behavioral outcomes. The within-subject design was selected to increase the statistical power of the study and maximize feasibility, as each participant serves as their own control. Given that psilocybin can produce strong acute subjective and long-lasting antidepressant effects, the study had a fixed-order design of placebo first, followed by psilocybin, in order to limit functional unblinding and minimize potential carryover effects. The two dosing sessions were conducted approximately four weeks apart and were embedded within an eight-session psychotherapy protocol (see Fig. 1 for study design). Participants were assessed for a total of 16 weeks. For a full discussion of blinding procedures, the psychotherapy protocol, and outcomes related to depression, anxiety and quality of life, see^11^.

### Study participants

Inclusion criteria included: (1) 18–65 years old; (2) Meet DSM-5 criteria for Major Depressive Disorder (MDD) by Structured Clinical Interview for DSM-5 (SCID-5-CT)^56^; (3) current moderate to severe major depressive episode of at least six weeks duration prior to screening, defined by a score ≥ 17 on the GRID-HAM-D-17^57,58^; (4) failed at least one adequate antidepressant trial (at least 6 weeks on a therapeutic dose) during the current depressive episode. Since medications commonly used in this population (e.g., serotonergic antidepressants) can interfere with the effects of psilocybin^59^, participants were required to be off any conventional antidepressant or antipsychotic medications for at least 2 weeks prior to study enrollment (or four weeks for fluoxetine). Exclusion criteria included: (1) primary psychiatric diagnosis other than MDD; (2) active substance use disorders; (3) past personal or family history of psychotic or bipolar disorder; (4) significant or unstable medical or neurological disease; (5) psilocybin exposure within the past year. For detailed inclusion/exclusion criteria see^11^.

### Recruitment and screening procedures

Participants were recruited primarily through online postings, including clinicaltrials.gov, clinician referrals and word of mouth. Interested candidates were prescreened by telephone and if eligible, underwent an in-depth screening process. This included a structured psychiatric assessment (SCID-5-CT), psychiatric history and evaluation, and medical screening. Informed consent was obtained from all study participants. Recruitment began in fall of 2018 and concluded in spring 2021.

### Interventions

#### Drugs

All dosing sessions were conducted at the VA Connecticut Healthcare System, West Haven, CT, USA. At the first dosing session, participants received placebo (microcrystalline cellulose) in an opaque capsule, while during the second session they received an identical capsule containing psilocybin (0.3 mg/kg, maximum dose 35 mg). While lower than the 30 mg/70 kg protocol for which safety has been demonstrated, this dose is sufficient for producing psychedelic effects^60^. Psilocybin was obtained from the University of Wisconsin and the Usona Institute. For detailed descriptions of drug administration sessions and procedures used to enhance blinding and minimize expectancy effects, see^11^.

#### Psychotherapeutic support

Participants were assigned a study therapist and psychiatrist for the entire duration of the study. An equivalent number and type of therapy sessions were provided before and after each of the two dosing sessions during the period of primary outcome collection (through week 6): A two-hour long preparatory psychotherapy/psychoeducation session preceded each dosing session, while two one-hour long debriefing/integration psychotherapy sessions were conducted 1 day and 1 week after each dosing session. Following collection of the final primary outcome measures at week 6, participants had two additional integration sessions to help sustain any initial clinical improvements. A delineated therapeutic framework and therapy manual was used to improve consistency in therapeutic approach across participants and between study therapists. This therapy protocol incorporated principles and elements of Acceptance and Commitment Therapy (ACT) (see^11^ for complete therapy manual). The rationale for including a specific psychotherapy and selecting ACT, the basic structure of the treatment model, and limitations to this approach, are described in detail separately^20^.

#### Outcome measures

Psychological flexibility was assessed using the Acceptance and Action Questionnaire-II (AAQ-II)^61^. Mindfulness was assessed using the Kentucky Inventory of Mindfulness Skills (KIMS)^62^, which includes the following scales: (a) Observe; (b) Describe; (c) Awareness; and (d) Accept Without Judgment. The degree to which participants are living in accordance with their values (values-congruent living) was measured with the Valued Living Questionnaire (VLQ)^63^. The AAQ-II, KIMS and VLQ were collected at baseline, week 2 (2 weeks after placebo), week 6 (2 weeks after psilocybin), and week 16 (3 months after psilocybin). Depression symptoms were assessed using the Quick Inventory of Depressive Symptomatology–Self-Report (QIDS-SR-16)^64^, which was collected throughout the study, from baseline (day before placebo) through week 16. Adverse events were monitored and recorded throughout the trial. Vital signs were monitored throughout each dosing session. For safety data along with other clinical and subjective measures collected, see^11^.

### Statistical analysis

The parent study from which data presented here was derived was powered primarily to investigate an electrophysiological biomarker of neuroplasticity rather than to demonstrate changes in clinical or behavioral outcomes. Data analysis was conducted on participants who completed at least one dosing session (ITT population). Data was analyzed using linear mixed models. Analysis of clinical outcomes through week 16 utilized time as a within-subjects factor. In order to explore the relationship between changes in depressive symptoms (QIDS-SR-16) and both (a) psychological flexibility (AAQ-II) and (b) Accept Without Judgment (KIMS), we conducted Spearman correlations between changes in these variables from before to 2 weeks after each dose. While the parent study also measured depression symptoms using the GRID-HAM-D-17, correlations in this substudy used the QIDS-SR-16 due to more available data points. Given the exploratory nature of the study and small sample size, all statistical tests were conducted using a two-tailed alpha threshold of 0.05, with no adjustments for multiple testing. All tests were performed using SAS, version 9.4 (SAS Institute Inc).

## Results

Of 949 individuals assessed for eligibility, 42 (4.4%) were screened in-person, and 22 (2.3%) were ultimately enrolled. 19 participants completed at least one dosing session, of which 15 completed both dosing sessions. Participant demographics and baseline clinical characteristics are presented in Table 1. The primary clinical outcomes related to depression, as well as anxiety and quality of life have been reported separately (see^11^).

**Table 1:** Demographics and baseline clinical characteristics (n = 19).

Age (years), mean (SD)	42.79 (13.8)
Age range (years)	20–61
Gender, n (%)	
Female	13 (68.4)
Male	6 (31.5)
Education level, n (%)	
High School	2 (10.5)
Some college	1 (5.2)
College	11 (57.8)
Masters	5 (26.3)
Marital/Relationship status, n (%)	
Married	7 (36.8)
Domestic partnership	2 (10.5)
Single	10 (52.6)
Employment Status, n (%)	
Unemployed	3 (15.7)
Full-time employed	7 (36.8)
Part-time employed	9 (47.3)
Race, n (%)	
White	16 (84.2)
Black	2 (10.5)
Two or more race	1 (5.2)
Ethnic origin, n (%)	
Hispanic	2 (10.5)
Non-Hispanic	17 (89.4)
Time since MDD diagnosis (years)/Estimated illness duration (years), mean (SD)	20 (12.0)
Previous antidepressant medication trials, mean (SD)	4.68 (1.9)
QIDS-SR^a^ score at screening, mean (SD)	17.05 (3.53)
Comorbid Psychiatric Conditions, n (%)	
Anxiety Disorders	11 (57.8)
Persistent depressive disorder	7 (36.8)
ADHD^b^	2 (10.5)
CPTSD^c^	2 (10.5)
Body dysmorphic disorder	1 (5.2)
Prior psychedelic use, n (%)	
Any	8 (42.1)
Psilocybin	7 (36.8)
LSD^d^	5 (26.3)
MDMA^e^	2 (10.5)
Time since last use of hallucinogens range (years)	3–24

a Quick Inventory of Depressive Symptomatology Self-Report. b Attention-Deficit/Hyperactivity Disorder. c Complex posttraumatic stress disorder. d Lysergic acid diethylamide. e Methylenedioxymethamphetamine.

### Psychological flexibility

**Figure 2:**
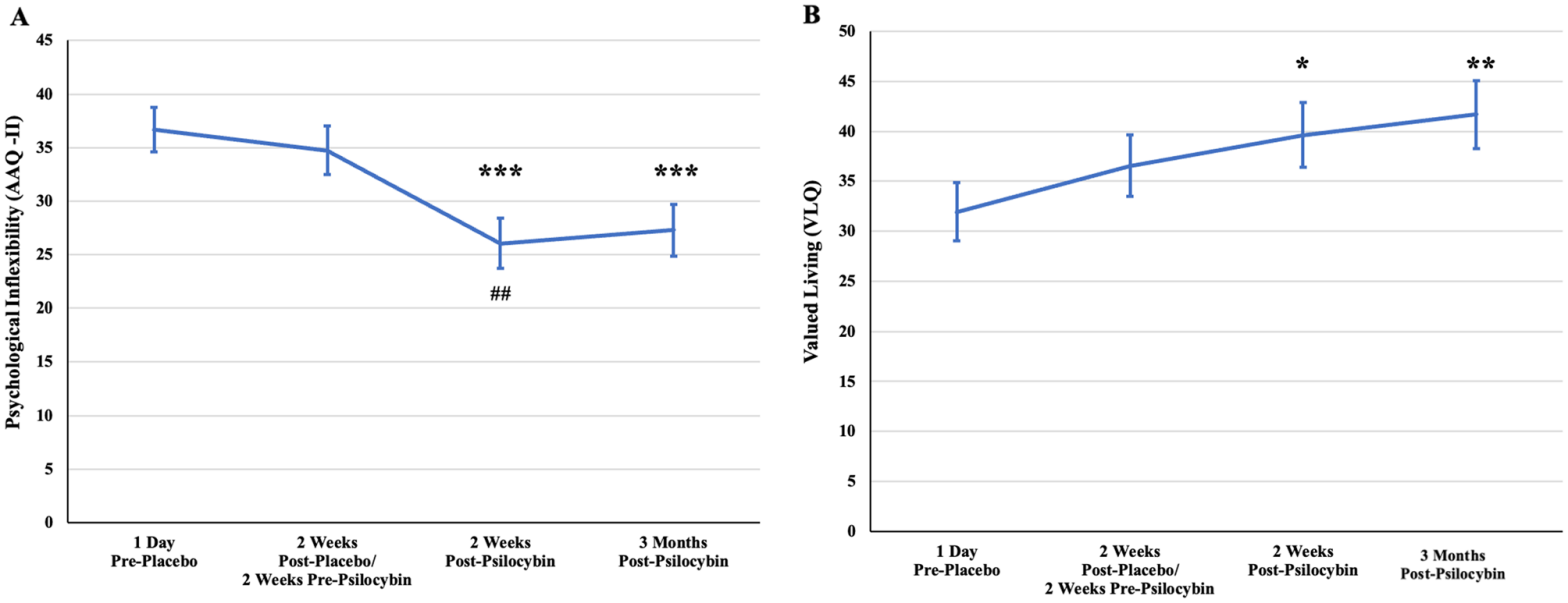
Changes in psychological inflexibility and valued living. (**A**) Significant reductions in psychological inflexibility 2 weeks post-psilocybin relative to baseline and 2 weeks pre-psilocybin. (**B**) Significant increases in valued living at 2 weeks and 4 weeks post-psilocybin relative to baseline. *Note.* Statistical significance relative to baseline = *p < .05, **p < .01, ***p < .001. Statistical significance relative to previous time point = ^##^*p* < .01.

AAQ-II scores decreased over time (i.e., increased psychological flexibility) (F_(3,35)_ = 8.74, *p* < 0.001) and were significantly lower following psilocybin administration compared to post-placebo (see Fig. 2 and Table 2). The significant reduction in AAQ-II scores was maintained through week 16.

**Table 2:** Changes in psychological flexibility, mindfulness, and values-congruent living.

Variable	Overall time effect	Mean (SE) change 2 weeks after placebo	Mean (SE) change 2 weeks after psilocybin	Difference (SE)
Acceptance and action questionnaire-II (AAQ-II)	F_(3,35)_ = 8.74, *p* < .001	1.94 (2.44), *p* = 0.433	10.64 (2.50), *p* = 0.0002	8.70 (2.46), *p* = 0.001
Valued living questionnaire (VLQ)	F_(3,36)_ = 2.99, *p* = 0.04	4.60 (3.32), *p* = 0.155	7.70 (3.32), *p* = 0.026	3.10 (3.27), *p* = 0.35
Observe (KIMS)	F_(3,36)_ = 0.72, *p* = 0.548	0.44 (1.41), *p* = 0.757	1.97 (1.49), *p* = 0.194	1.53 (1.41), *p* = 0.287
Awareness (KIMS)	F_(3,36)_ = 2.02, *p* = 0.129	1.18 (1.35), *p* = 0.39	3.37 (1.41), *p* = 0.023	2.20 (1.38), *p* = 0.121
Describe (KIMS)	F_(3,36)_ = 2.87, *p* = 0.05	1.41 (1.02), *p* = 0.176	2.63 (1.08), *p* = 0.02	1.22 (1.02), *p* = 0.237
Accept without judgment (KIMS)	F_(3,36)_ = 3.38, *p* = 0.029	3.38 (1.79), *p* = 0.068	5.81 (1.89), *p* = 0.004	2.43 (1.81), p = 0.188

### Values-congruent living

VLQ scores increased over time (i.e., improvement in values-congruent living) (F_(3,36)_ = 2.99, *p* = 0.044) and were significantly increased 2 weeks following psilocybin administration compared to baseline but not compared to post-placebo (see Fig. 2 and Table 2). The increases in VLQ scores remained significant through week 16. Scores were not significantly improved following placebo.

### Mindfulness

**Figure 3:**
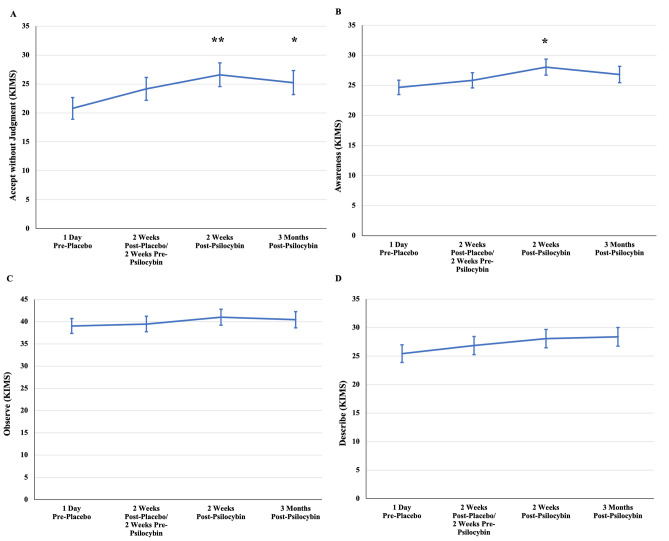
Changes in mindfulness domains. (**A**) Significant increases in Acceptance Without Judgment at 2 weeks and 4 weeks post-psilocybin relative to baseline. (**B**) Significant increases in Awareness at 2 weeks post-psilocybin relative to baseline. (**C**) No significant changes in Observe. (**D**) Significant increases in Describe at 2 weeks and 4 weeks post-psilocybin relative to baseline. *Note.* Statistical significance relative to baseline = *p < .05, **p < .01, ***p < .001. Statistical significance relative to previous time point = ^##^*p* < .01.

Time analyses were conducted for each of the 4 subscales of the KIMS (see Fig. 3 and Table 2). Significant increases (i.e., improvements) were observed on the Describe (F_(3,36)_ = 2.87, *p* = 0.05) and Accept Without Judgment subscales (F_(3,36)_ = 3.38, *p* = 0.029). Describe and Accept Without Judgment were significantly increased 2 weeks following psilocybin administration compared to baseline but not compared to post-placebo. The increased scores remained significant through week 16. Describe and Accept Without Judgment were not significantly improved following placebo. There were no significant changes in the Observe (F_(3,36)_ = 0.72, *p* = 0.548) or Awareness (F_(3,36)_ = 2.02, *p* = 0.129) scales.

### Relationship between changes in depression symptoms and changes in psychological flexibility and accept without judgment

Depression scores (QIDS-SR-16) decreased from 1 day before (mean = 16.79, SE = 0.95) to 2 weeks after (mean = 11.74, SE = 1.49) placebo (Δ mean = 5.05, *p* = 0.005). There was not a significant correlation between change in depression scores and change in either (a) psychological inflexibility (Spearman's rho = 0.30, *p* = 0.295) or (b) Accept Without Judgment (Spearman's rho = -0.42, *p* = 0.140) during this time period.

**Figure 4:**
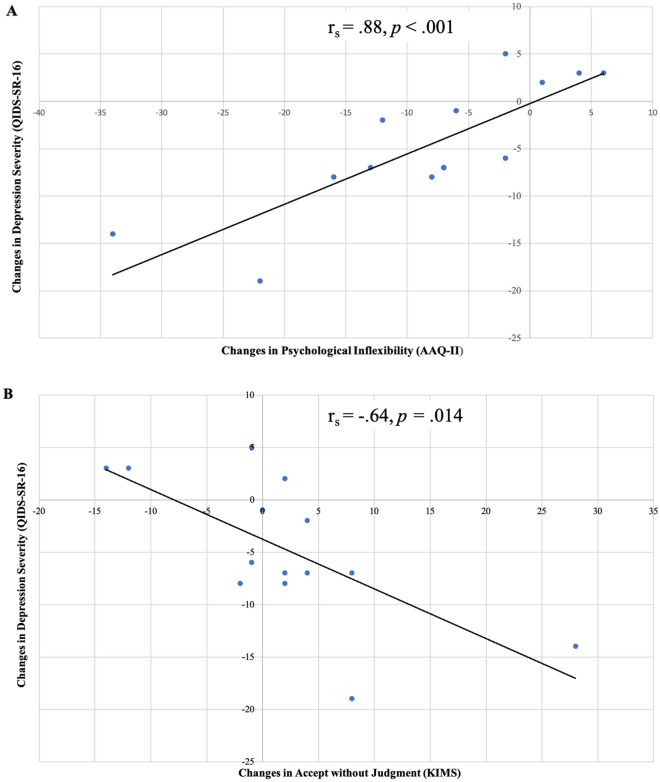
Association between post-psilocybin changes in depression severity and psychological inflexibility/Accept Without Judgment. (**A**) Significant positive association between reductions in psychological inflexibility and depression severity. (**B**) Significant negative association between increases in Acceptance Without Judgment inflexibility and reductions in depression severity.

Depression scores decreased from 2 weeks before (mean = 11.71, SE = 1.49) to 2 weeks after (mean = 6.27, SE = 1.38) psilocybin (Δ mean = 5.47*, p* = 0.016). Reductions in depression scores were significantly associated with (a) decreases in psychological inflexibility (Spearman's rho = 0.88, *p* < 0.001) and (b) increases in Accept Without Judgment (Spearman's rho = -0.64, *p* = 0.014) during this time period (Fig. 4).

## Discussion

This placebo-controlled, within-subject, fixed-order trial among participants with long-standing and predominantly treatment-resistant major depression documented significant and durable improvements in measures of psychological flexibility, mindfulness, and valued living following administration of both placebo and a moderate dose of psilocybin in combination with ACT-based psychotherapy. Improvements in psychological flexibility were significantly greater 2 weeks after psilocybin compared to 2 weeks after placebo and were maintained for 3 months after the psilocybin dosing session. Moreover, following psilocybin administration, improvements in measures of psychological flexibility (AAQ-II) and experiential acceptance (KIMS, Accept Without Judgment scale) were significantly correlated with reductions in depression severity.

The significant improvements in psychological flexibility observed post-psilocybin are in line with the aim of the study design to integrate psilocybin dosing sessions within a psychotherapeutic platform that specifically targets psychological flexibility. These results are consistent with previous naturalistic research indicating that classic psychedelic use is associated with improvements in psychological flexibility^21,36,65^ and are the first to extend this finding to psilocybin or a clinical trial specifically. Given the significant improvement in psychological flexibility post-psilocybin compared to post-placebo, as well as the finding that values-congruent living and mindfulness domains only significantly improved following psilocybin dosing, our data suggests that the psilocybin dosing session had a significant impact on these psychological domains. In other words, the results suggest that improvements in psychological flexibility are likely not attributable exclusively to the ACT-based psychotherapeutic platform or placebo effects. However, carryover effects from the placebo phase of the study are a potential contributing factor given the within subject study design.

Additionally, significant increases were found over time for values-congruent living and in two domains of mindfulness (Acceptance Without Judgment and Describe). While these increases only became significant following psilocybin dosing, they were not significantly greater post-psilocybin compared to post-placebo. These results may be attributable to several factors, including the ACT-based therapeutic intervention itself being the cause of improvements in these domains, minimal power to detect psilocybin specific effects, and issues with the fixed-order design of the study (e.g. carryover effects, gains occurring in the early phase of treatment^66^). Of note, no significant overall time effect was observed for the mindfulness domains of Observe and Awareness. These findings are in line with previous research that has found significant improvement in experiential acceptance following psilocybin-assisted therapy^5^ and mindful non-judgment following the administration of psilocybin but no significant changes in other facets of mindfulness following the administration of psilocybin^50^.

The significant associations between reductions in depression severity and increases in psychological flexibility and experiential acceptance support the notion that changes in psychological flexibility and related concepts of mindfulness and acceptance may be important psychological mechanisms of change in psilocybin-assisted therapy of depression, and perhaps, other disorders. These findings are especially notable given that the parent clinical trial did not find a significant correlation between the strength of mystical-type experience during psilocybin dosing and reductions in depression^11^. While it is possible that a small sample size and limited variability in MEQ scores during psilocybin dosing contributed to the latter finding, it is also possible that there may be more essential acute experiences (e.g., psychological insight, emotional breakthrough) and post-acute changes (e.g., increases in psychological flexibility and experiential acceptance) that predict psilocybin treatment outcomes. It is important to note that the correlation between reductions in depression severity and increases in psychological flexibility do not necessarily imply causation and it is possible that improvements in depression also lead to greater psychological flexibility. Nonetheless, these findings are consistent with past survey-based, observational, and clinical research linking post-psychedelic increases in psychological flexibility^21,36–38,65^ and experiential acceptance^22,41^ with improvements in depression severity and a range of mental health outcomes.

### Limitations and future directions

An important limitation of this within-subject study is the inability to fully disambiguate the relative contribution of each therapeutic intervention (placebo dosing, psilocybin dosing, and ACT-based psychotherapy) to the results observed. Because of this, as well as the possibility of carryover effects from the placebo phase, the persisting psychological changes observed following psilocybin dosing cannot be definitively attributed specifically to psilocybin. Moreover, given that we did not compare ACT-based therapy to another therapeutic approach, it is unclear if similar results would have been observed if the therapists had used a non-ACT-based model (e.g. psychological support) to guide preparation and integration. Future studies may attempt to compare the relative efficacy of different therapeutic modalities or the amount of psychotherapeutic support provided.

Furthermore, findings from the present study cannot firmly establish a causal effect of psilocybin therapy on psychological flexibility (for further discussion, see^67^). Additional research remains necessary to elucidate the potential mechanistic role of psychological flexibility within psilocybin therapy. For instance, intensive longitudinal designs (e.g., the use of ecological momentary assessment) may help to further elucidate the temporal relationship between changes in psychological flexibility and depressive symptoms. Similarly, dose–response studies with variable dosages of psilocybin and amounts of psychotherapy may also help to establish the gradient of the relationship between psychological flexibility and depressive symptoms within psilocybin therapy. Future research examining the effects of psilocybin-assisted therapy on psychological flexibility (and its subdomains) should also include alternative measures of psychological flexibility (e.g., the Multidimensional Psychological Flexibility Inventory^68^), given significant concerns regarding the validity of the AAQ-II as a measure of psychological flexibility^69,70^, including its overlap with measures of distress and its single factor measurement of psychological flexibility.

Finally, findings from the present study should be interpreted in light of difficulties with blinding and the likely contribution of expectancy effects (for in depth discussion, see^11,71^). This issue remains ubiquitous across psychedelic research^72^ and is likely a problem more broadly within psychiatry (e.g., see^73^).

## Conclusion

In conclusion, this placebo-controlled, within-subject, fixed-order trial of psilocybin-assisted therapy for MDD demonstrated significant improvements in psychological flexibility, several facets of mindfulness (accept without judgment and describe, but not observe and awareness) and values-congruent living. Improvements in psychological flexibility (but not mindfulness or values-congruent living) were greater post-psilocybin compared to post-placebo. Additionally, improvements in psychological flexibility and experiential acceptance were strongly associated with reductions in depression severity. These findings support the theoretical premise of integrating psilocybin dosing sessions with psychotherapeutic platforms that target psychological flexibility and add to the emerging evidence that psychological flexibility may be an important putative mechanism of change in psilocybin-assisted therapy for MDD and potentially, other mental health conditions.

## Data Availability

The datasets generated during and/or analyzed during the current study are available from the corresponding author on reasonable request.

## References

[1] Johnson MW, Hendricks PS, Barrett FS, Griffiths RR (2019). Classic psychedelics: An integrative review of epidemiology, therapeutics, mystical experience, and brain network function. Pharmacol. Ther..

[2] Schimmers N (2022). Psychedelics for the treatment of depression, anxiety, and existential distress in patients with a terminal illness: a systematic review. Psychopharmacology.

[3] Vollenweider FX, Vollenweider-Scherpenhuyzen MF, Babler A, Vogel H, Hell D (1998). Psilocybin induces schizophrenia-like psychosis in humans via a serotonin-2 agonist action. Neuroreport.

[4] Madsen MK (2019). Psychedelic effects of psilocybin correlate with serotonin 2A receptor occupancy and plasma psilocin levels. Neuropsychopharmacology.

[5] Carhart-Harris R (2021). Trial of psilocybin versus escitalopram for depression. New Engl. J. Med..

[6] von Rotz R (2023). Single-dose psilocybin-assisted therapy in major depressive disorder: A placebo-controlled, double-blind, randomised clinical trial. EClinicalMedicine.

[7] Goodwin GM (2022). Single-dose psilocybin for a treatment-resistant episode of major depression. N. Engl. J. Med..

[8] Bogenschutz MP (2022). Percentage of heavy drinking days following psilocybin-assisted psychotherapy vs placebo in the treatment of adult patients with alcohol use disorder: A randomized clinical trial. JAMA Psychiatry.

[9] Davis AK (2021). Effects of psilocybin-assisted therapy on major depressive disorder. JAMA Psychiatry.

[10] D'Souza DC (2022). Exploratory study of the dose-related safety, tolerability, and efficacy of dimethyltryptamine (DMT) in healthy volunteers and major depressive disorder. Neuropsychopharmacology.

[11] Sloshower J (2023). Psilocybin-assisted therapy for major depressive disorder: An exploratory placebo-controlled, fixed-order trial. J. Psychopharmacol..

[12] Griffiths RR (2016). Psilocybin produces substantial and sustained decreases in depression and anxiety in patients with life-threatening cancer: A randomized double-blind trial. J. Psychopharmacol..

[13] Ross S (2016). Rapid and sustained symptom reduction following psilocybin treatment for anxiety and depression in patients with life-threatening cancer: A randomized controlled trial. J. Psychopharmacol..

[14] Carhart-Harris RL (2016). Psilocybin with psychological support for treatment-resistant depression: an open-label feasibility study. Lancet Psychiatry.

[15] Gukasyan N (2022). Efficacy and safety of psilocybin-assisted treatment for major depressive disorder: Prospective 12-month follow-up. J. Psychopharmacol..

[16] Agin-Liebes GI (2020). Long-term follow-up of psilocybin-assisted psychotherapy for psychiatric and existential distress in patients with life-threatening cancer. J. Psychopharmacol..

[17] Garcia-Romeu A, Griffiths RR, Johnson MW (2014). Psilocybin-occasioned mystical experiences in the treatment of tobacco addiction. Curr. Drug Abuse Rev..

[18] Bogenschutz MP (2015). Psilocybin-assisted treatment for alcohol dependence: A proof-of-concept study. J. Psychopharmacol..

[19] Skosnik PD (2023). Sub-acute effects of psilocybin on EEG correlates of neural plasticity in major depression: Relationship to symptoms. J. Psychopharmacol..

[20] Sloshower J (2020). Psilocybin-assisted therapy of major depressive disorder using acceptance and commitment therapy as a therapeutic frame. J. Context. Behav. Sci..

[21] Davis AK, Barrett FS, Griffiths RR (2020). Psychological flexibility mediates the relations between acute psychedelic effects and subjective decreases in depression and anxiety. J. Context. Behav. Sci..

[22] Zeifman RJ (2020). Post-psychedelic reductions in experiential avoidance are associated with decreases in depression severity and suicidal ideation. Front. Psychiatry.

[23] Carhart-Harris RL, Erritzoe D, Haijen E, Kaelen M, Watts R (2018). Psychedelics and connectedness. Psychopharmacology.

[24] Watts R (2022). The watts connectedness scale: A new scale for measuring a sense of connectedness to self, others, and world. Psychopharmacology.

[25] Carhart-Harris RL, Friston KJ (2019). REBUS and the Anarchic Brain: Toward a unified model of the brain action of psychedelics. Pharmacol. Rev..

[26] Zeifman, R. J. *et al.* From relaxed beliefs under psychedelics (rebus) to revised beliefs after psychedelics (rebas): Preliminary development of the relaxed beliefs questionnaire (reb-q). (2022). 10.1038/s41598-023-28111-3 PMC11779827 39881126

[27] Roseman L (2019). Emotional breakthrough and psychedelics: Validation of the Emotional Breakthrough Inventory. J. Psychopharmacol..

[28] Hayes SC, Pistorello J, Levin ME (2012). Acceptance and commitment therapy as a unified model of behavior change. Counsel. Psychol..

[29] Francis AW, Dawson DL, Golijani-Moghaddam N (2016). The development and validation of the Comprehensive assessment of Acceptance and Commitment Therapy processes (CompACT). J. Context. Behav. Sci..

[30] Stange JP, Alloy LB, Fresco DM (2017). Inflexibility as a vulnerability to depression: A systematic qualitative review. Clin. Psychol. Sci. Pract..

[31] Akbari M, Seydavi M, Hosseini ZS, Krafft J, Levin ME (2022). Experiential avoidance in depression, anxiety, obsessive-compulsive related, and posttraumatic stress disorders: A comprehensive systematic review and meta-analysis. J. Context. Behav. Sci..

[32] Forman EM, Herbert JD, Moitra E, Yeomans PD, Geller PA (2007). A randomized controlled effectiveness trial of acceptance and commitment therapy and cognitive therapy for anxiety and depression. Behav. Modif..

[33] Ostergaard T, Lundgren T, Zettle RD, Landro NI, Haaland VO (2020). Psychological flexibility in depression relapse prevention: Processes of change and positive mental health in group-based act for residual symptoms. Front. Psychol..

[34] Stockton D (2019). Identifying the underlying mechanisms of change during acceptance and commitment therapy (ACT): A systematic review of contemporary mediation studies. Behav. Cogn. Psychother.

[35] Walser RD (2015). Effectiveness of acceptance and commitment therapy in treating depression and suicidal ideation in Veterans. Behav. Res. Ther..

[36] Close JB, Hajien EC, Watts R, Roseman L, Carhart-Harris RL (2020). Psychedelics and psychological flexibility–Results of a prospective web-survey using the Acceptance and Action Questionnaire II. J. Context. Behav. Sci..

[37] Agin-Liebes G (2022). Prospective examination of the therapeutic role of psychological flexibility and cognitive reappraisal in the ceremonial use of ayahuasca. J. Psychopharmacol..

[38] González D (2020). Therapeutic potential of ayahuasca in grief: A prospective, observational study. Psychopharmacology.

[39] Cardaciotto L, Herbert JD, Forman EM, Moitra E, Farrow V (2008). The assessment of present-moment awareness and acceptance: The Philadelphia Mindfulness Scale. Assessment.

[40] Fletcher L, Hayes SC (2005). Relational frame theory, acceptance and commitment therapy, and a functional analytic definition of mindfulness. J. Rational-Emot. Cognit. Behav. Therapy.

[41] Zeifman RJ, Wagner AC, Monson CM, Carhart-Harris RL (2023). How does psilocybin therapy work? An exploration of experiential avoidance as a putative mechanism of change. J. Affect. Disord..

[42] Dominguez-Clave E (2019). Ayahuasca improves emotion dysregulation in a community sample and in individuals with borderline-like traits. Psychopharmacology.

[43] Murphy-Beiner A, Soar K (2020). Ayahuasca's 'afterglow': Improved mindfulness and cognitive flexibility in ayahuasca drinkers. Psychopharmacology.

[44] Sampedro F (2017). Assessing the psychedelic "after-glow" in Ayahuasca users: Post-acute neurometabolic and functional connectivity changes are associated with enhanced mindfulness capacities. Int. J. Neuropsychopharmacol..

[45] Soler J (2016). Exploring the therapeutic potential of Ayahuasca: Acute intake increases mindfulness-related capacities. Psychopharmacology.

[46] Soler J (2018). Four weekly ayahuasca sessions lead to increases in "acceptance" capacities: A comparison study with a standard 8-week mindfulness training program. Front. Pharmacol..

[47] Uthaug MV (2020). Prospective examination of synthetic 5-methoxy-N, N-dimethyltryptamine inhalation: effects on salivary IL-6, cortisol levels, affect, and non-judgment. Psychopharmacology.

[48] Uthaug MV (2019). A single inhalation of vapor from dried toad secretion containing 5-methoxy-N, N-dimethyltryptamine (5-MeO-DMT) in a naturalistic setting is related to sustained enhancement of satisfaction with life, mindfulness-related capacities, and a decrement of psychopathological symptoms. Psychopharmacology.

[49] Uthaug MV (2018). Sub-acute and long-term effects of ayahuasca on affect and cognitive thinking style and their association with ego dissolution. Psychopharmacology.

[50] Kiraga MK, Kuypers KP, Uthaug MV, Ramaekers JG, Mason NL (2022). Decreases in state and trait anxiety post-psilocybin: A naturalistic, observational study among retreat attendees. Front. Psychiatry.

[51] Madsen MK (2020). A single psilocybin dose is associated with long-term increased mindfulness, preceded by a proportional change in neocortical 5-HT2A receptor binding. Eur. Neuropsychopharmacol..

[52] Smigielski L (2019). Characterization and prediction of acute and sustained response to psychedelic psilocybin in a mindfulness group retreat. Sci. Rep..

[53] Radakovic C, Radakovic R, Peryer G, Geere J-A (2022). Psychedelics and mindfulness: A systematic review and meta-analysis. J. Psychedelic Stud..

[54] Johnson MW, Garcia-Romeu A, Johnson PS, Griffiths RR (2017). An online survey of tobacco smoking cessation associated with naturalistic psychedelic use. J. Psychopharmacol..

[55] Griffiths RR (2018). Psilocybin-occasioned mystical-type experience in combination with meditation and other spiritual practices produces enduring positive changes in psychological functioning and in trait measures of prosocial attitudes and behaviors. J. Psychopharmacol..

[56] First, M., Williams, J., Karg, R. & Spitzer, R. *Structured Clinical Interview for DSM-5 Disorders, Clinical Trials Version (SCID-5-CT)*. (American Psychiatric Association, 2015).

[57] Hamilton M (1960). A rating scale for depression. J. Neurol. Neurosurg. Psychiatry.

[58] International Society for CNS Drug Development. *GRID-HAMD-17 Structured Interview Guide*. (ISCDD, 2003).

[59] Malcolm B, Thomas K (2021). Serotonin toxicity of serotonergic psychedelics. Psychopharmacology.

[60] Griffiths RR, Richards WA, McCann U, Jesse R (2006). Psilocybin can occasion mystical-type experiences having substantial and sustained personal meaning and spiritual significance. Psychopharmacology.

[61] Bond FW (2011). Preliminary psychometric properties of the acceptance and action questionnaire–II: A revised measure of psychological inflexibility and experiential avoidance. Behav. Therapy.

[62] Baer RA, Smith GT, Allen KB (2004). Assessment of mindfulness by self-report: The Kentucky Inventory of Mindfulness Skills. Assessment.

[63] Wilson KG, Sandoz EK, Kitchens J, Roberts M (2010). The Valued Living Questionnaire: Defining and measuring valued action within a behavioral framework. Psychol. Record.

[64] Rush AJ (2003). The 16-Item Quick Inventory of Depressive Symptomatology (QIDS), clinician rating (QIDS-C), and self-report (QIDS-SR): A psychometric evaluation in patients with chronic major depression. Biol. Psychiatry.

[65] Davis AK, Xin Y, Sepeda ND, Garcia-Romeu A, Williams MT (2021). Increases in psychological flexibility mediate relationship between acute psychedelic effects and decreases in racial trauma symptoms among people of color. Chronic Stress.

[66] Shalom JG, Aderka IM (2020). A meta-analysis of sudden gains in psychotherapy: Outcome and moderators. Clin. Psychol. Rev..

[67] Kangaslampi S (2020). Uncovering psychological mechanisms mediating the effects of drugs: Some issues and comments using the example of psychedelic drugs. Psychopharmacology.

[68] Rolffs JL, Rogge RD, Wilson KG (2018). Disentangling components of flexibility via the Hexaflex model: Development and validation of the multidimensional psychological flexibility inventory (MPFI). Assessment.

[69] Tyndall I (2019). The acceptance and action questionnaire-II (AAQ-II) as a measure of experiential avoidance: Concerns over discriminant validity. J. Context. Behav. Sci..

[70] Doorley JD, Goodman FR, Kelso KC, Kashdan TB (2020). Psychological flexibility: What we know, what we do not know, and what we think we know. Social Personal. Psychol. Compass.

[71] Muthukumaraswamy S, Forsyth A, Lumley T (2021). Blinding and expectancy confounds in psychedelic randomised controlled trials. Expert Rev. Clin. Phar..

[72] Wen A (2023). A systematic review of study design and placebo controls in psychedelic research. Psychedel. Med..

[73] Scott AJ, Sharpe L, Colagiuri B (2022). A systematic review and meta-analysis of the success of blinding in antidepressant RCTs. Psychiatry Res..

